# Record linkage for Routinely Collected Health Data in an African Health Information Exchange.

**DOI:** 10.23889/ijpds.v7i3.2022

**Published:** 2022-08-25

**Authors:** Themba Mutemaringa, Alexa Heekes, Andrew Boulle, Nicki Tiffin

**Affiliations:** 1 Provincial Health Data Centre, Western Cape Government Health, South Africa; CIDER, University Of Cape Town

## Objectives

- To describe the record linkage system that is currently implemented at the Provincial Health Data Centre (PHDC) in the Western Cape, South Africa

To assess its output to date with respect to types of matches and duplicates trends

To describe the errors affecting patient matching

## Approach

We apply a stepwise deterministic record linkage approach to link patient data that are routinely collected from health information systems in the Western Cape province of South Africa. Variables used in the linkage process include South African National Identity number (RSA ID), date of birth, year of birth, month of birth, day of birth, residential address and contact information. Matching records are established from sequentially running the data through multiple passes formed by various combinations of linkage variables. Descriptive analyses are used to estimate the extent of mismatches and duplication in the provincial patient master index (PMI).

## Results

- The proportion of duplicates dropped from approximately 16.8% in December 2015 to 9.6% in October 2020, indicating improved data linkage over time.

Duplicates mainly arise from spelling errors, and surname and first names carry most of the errors, with different first names and surname for the same individual in approximately 22% of duplicates.

Linkage is also affected by completeness, with less than 30 % completeness for the South African national identity (RSA ID) number which is mainly because RSA ID is not mandatory when seeking healthcare.

## Conclusion

Linkage improvement could be due to improved registration practices. Further improvements are possible by repeating data linkage where patients register before creating a new patient record following a failed search. This could use the PHDC linkage approach whilst leveraging all data in addition to search terms used by the clerk.

